# The Effect of Vasopressin on 90-Day Mortality in Patients With Cardiogenic Shock: A Retrospective Cohort Study Using Propensity Score-Weighted Analysis

**DOI:** 10.1155/cdr/9920490

**Published:** 2025-05-28

**Authors:** Christophe Beyls, Thomas Hanquiez, Nicolas Mollet, Yoni Sarfati, Adel Zerima, Souheil Chafiki, Patricia Besserve, Hervé Dupont, Momar Diouf, Osama Abou-Arab, Yazine Mahjoub

**Affiliations:** ^1^Department of Anesthesiology and Critical Care Medicine, Amiens University Hospital, Amiens, France; ^2^UR UPJV 7518 SSPC (Simplification of Care of Complex Surgical Patients) Research Unit, University of Picardie Jules Verne, Amiens, France; ^3^Department of Cardiology, Amiens University Hospital, Amiens, France; ^4^Department of Statistics, Amiens University Hospital, Amiens, France

**Keywords:** cardiogenic shock, mortality, norepinephrine, vasopressin, vasopressor

## Abstract

**Background:** Cardiogenic shock (CS) may lead to a refractory vasoplegic state that requires vasopressin on top of norepinephrine. Vasopressin has been available in France since January 2022. However, data assessing the clinical impact of vasopressin in CS are very scarce.

**Objective:** In this study, we aimed to assess the association between vasopressin and 90-day mortality in a cohort of CS.

**Method:** We conducted a retrospective, single-center study at Amiens University Hospital comparing two cohorts of patients experiencing at least Stage C of CS: one historical cohort from 2018 to 2019 without vasopressin and a contemporary cohort from 2022 to 2023 treated with vasopressin. The primary outcome was 90-day mortality. The secondary outcome was the occurrence of serious adverse events (SAEs) during ICU stay. Inverse probability of treatment weighting (IPTW) derived from propensity score was used to reduce imbalances in baseline characteristics.

**Results:** We included 201 patients in the study: 59 in the vasopressin group and 142 in the no vasopressin group. The SOFA score and norepinephrine equivalent were higher in the vasopressin group (13 [10–16] vs. 12 [9–15]; *p* = 0.02 and 0.72 [0.21–1.51] vs. 0.13 [0.07–0.34]; *p* < 0.001, respectively). There was no significant difference between the two groups for the 90-day mortality (*n* = 31/59 vs. 75/142; *p* = 0.97). Before adjustment, vasopressin was not associated with 90-day mortality (OR = 0.98 [95% CI 0.50–1.78]; *p* = 0.87). After weighting, vasopressin remained not associated with 90-day mortality (OR = 1.10 [95% CI 0.56–2.17]; *p* = 0.77). There was no significant difference for SAEs between the two groups (*n* = 57/142 [40%] vs. *n* = 23/59 [39%]; *p* = 0.88).

**Conclusion:** Vasopressin was not associated with 30-day mortality and SAEs in patients with CS.

## 1. Background

Cardiogenic shock (CS), primarily stemming from acute myocardial infarction (accounting for over 80% of CS cases [1]), persists as a significant cause of mortality [2] despite recent advancements in therapeutic options [3]. CS represents a continuum that displays a broad spectrum of clinical presentations [4] associated with short-term mortality rates that vary considerably, ranging from 5% to 82% [1]. The variability in CS definitions across published studies [5, 6] has contributed to some confusion, particularly when assessing the association between pharmacological or mechanical support treatments and outcomes in patients with CS [3]. From a pathophysiological standpoint, CS is triggered by a severe reduction in myocardial performance, leading to decreased cardiac output, hemodynamic instability, pulmonary congestion-induced hypoxia, and end-organ hypoperfusion [7]. The hallmark of CS is peripheral vasoconstriction to achieve organ perfusion. Still, CS is frequently associated with a vasoplegic state characterized by a decrease in systemic vascular resistance, necessitating the administration of vasopressors such as norepinephrine (NE) [8]. In the case of refractory shock or for catecholamine sparing, vasoconstrictors acting through nonadrenergic cellular pathways can be utilized [8].

Vasopressin, a 9-amino acid endogenous hormone synthesized by the hypothalamus, exhibits vasopressor and osmoregulatory effects [9]. In response to hypotension, vasopressin promotes vasoconstriction through various pathways, including modulation of adenosine triphosphate-sensitive K+ channel function [10], nitric oxide production, and enhancement of the vascular response to vasoactive drugs [9]. Plasma vasopressin levels were disproportionately low in patients with vasodilatory shock [11]. Nonetheless, the administration of exogenous vasopressin exhibited a restoration of vasomotor responsiveness. Subsequently, numerous investigations have attempted to clarify the efficacy of vasopressin or its analogs in managing shock states characterized by profound vasodilation, including septic shock [12] and postcardiopulmonary bypass vasoplegic syndrome [13]. The results of these studies have shown that administering vasopressin improves clinical outcomes, thereby positioning vasopressin as an alternative to or in combination with NE [14].

However, some of these studies included patients with CS [13], raising questions about the effectiveness of vasopressin administration because plasma levels of vasopressin were higher in CS compared to septic shock [15]. To date, there is little evidence of vasopressin's clinical impact on CS patients.

We hypothesize that administering vasopressin in addition to the currently recommended vasoactive drugs for CS may improve the prognosis of these patients but may also increase the occurrence of serious adverse events (SAEs).

## 2. Methods

### 2.1. Creation of Cohorts

In this retrospective, single-center study, we included all adult patients (> 18 years of age) with at least Stage C of CS (Society for Cardiovascular Angiography and Interventions (SCAI) classification [7]) who were hospitalized in the cardiothoracic and vascular respiratory intensive care unit (ICU-CTVR) at the Amiens-Picardie University Hospital. Exclusion criteria were patients for whom the diagnosis of CS was ruled out in favor of alternative diagnoses, such as septic shock, hemorrhagic shock, and refractory cardiac arrest. Additionally, terminally ill patients, individuals under legal guardianship or conservatorship, subjects with significant missing data, and those lost to follow-up were also excluded. To analyze the impact of vasopressin, we compared two groups of CS patients: the vasopressin group and the no vasopressin group.

*The vasopressin group*: Patients in the vasopressin group were included based on a prospective cohort of consecutive CS patients who received vasopressin in addition to NE and dobutamine between January 2022 and July 2023. We excluded data from the intervening period of 2019–2021 due to the COVID-19 epidemic. In our center, vasopressin can be administered if NE exceeds 0.2 *μ*g/kg/min. Patients were enrolled in the vasopressin group on the day of administration.

*No vasopressin group*: The no vasopressin groups were based on a “historical” cohort of CS patients treated between January 2018 and July 2019, during which vasopressin was unavailable in our center. We attempted to retrospectively select patients who would meet the current criteria for vasopressin prescription according to our department protocol. In the no vasopressin group, patients were enrolled with a minimum dose of 0.2 *μ*g/kg/min of NE along with dobutamine administration.

### 2.2. CS Definition

We included patients who met the criteria for at least Stage C of CS according to the SCAI classification [7]. Stage C, often referred to as the “classic” stage of CS, was characterized by hypoperfusion necessitating a series of initial interventions beyond volume resuscitation to restore adequate perfusion. These interventions included administration of vasoactive agents, positive pressure ventilation, mechanical support, and/or extracorporeal membrane oxygenation (ECMO). These criteria align with the most recent guidelines provided by the SCAI classification. Patients admitted to our center with Stages D and E of CS were also enrolled in the study.

### 2.3. Ethics

The study followed the Declaration of Helsinki and was approved by the Institutional Review Board of Amiens University Hospital (Number: PI2023-843-0133) for noninterventional studies involving humans. Informed consent was waived by French law on clinical research for noninterventional studies [16]. The report was in accordance with the guidelines on reports for propensity score analysis [17] and STROBE statement for retrospective studies [18].

### 2.4. Outcomes

*The primary outcome* was 90-day all-cause mortality, defined as death from any cause occurring within 90 days following inclusion in the study.

*The secondary outcomes* included the occurrence of SAEs, which were determined by the presence of one of the following events: digestive ischemia (comprising acute mesenteric ischemia and/or ischemic colitis), digital ischemia, and ST-segment elevation myocardial infarction (STEMI) as confirmed by a 12-lead electrocardiogram. Additionally, we assessed the dose of NE equivalent by NEE score [19]. Hence, NEE score was calculated using the following formula without including vasopressin: NEE score = NE dose (*μ*g/min) + epinephrine dose (*μ*g/min) + 1/100 × dopamine dose (*μ*g/min) + 1/2.2 × phenylephrine dose (*μ*g/kg/min) [19]. In our center, we used NE bitartrate [20].

### 2.5. Follow-Up

Follow-up was conducted using information from our institution's medical records, input from referring cardiologists, and phone conversations with patients or their family members. We ensured a comprehensive follow-up rate of 100%.

### 2.6. Data Collection

Clinical, biological, echocardiographic, and operative data were collected retrospectively for the historical cohort and prospectively for the vasopressin cohort in a computerized database. In our department, prescription and administration of therapies are computerized. Changes in the infusion rate of vasopressors are electronically documented and reported in our software. We performed computerized data extraction from our local data systems, DxCare (DxCare, Medasys), Diane Rea (Bow Medical, France), and Clinisoft (Centricity Critical Care Clinisoft, GE Healthcare).

The following clinical data were collected: age, gender, height, and weight for calculating body mass index (BMI), cardiovascular risk factors, history of cardiovascular and chronic diseases, and medications taken at home. The following biological data were collected: arterial blood gas parameters, including pH, lactate levels, troponin, creatinine, and hemoglobin, collected on Days 0, 1, 2, and 3. Doses of catecholamine were recorded via NEE score and vaso-inotropic score (VIS) [21] on Days 0, 1, 2, and 3. The sepsis organ failure assessment (SOFA) score was calculated at inclusion (Day 0) [22].

### 2.7. Statistical Analysis

Categorical variables were presented as frequencies and percentages, and comparisons between the two groups were made using the *χ*^2^ or Fisher's exact test, as appropriate. Continuous variables were expressed as mean ± standard deviation and compared using unpaired Student's *t*-test or the Mann–Whitney test when appropriate.

### 2.8. Missing Data and Propensity Score

Missing data were imputed using predictive mean matching imputation, resulting in the creation of five imputed datasets. Following the Rubin rule [23], missing data were treated as missing-not-at-random. For the primary outcome of 90-day all-cause mortality, prognostic variables associated with a 10% significance level in univariate analysis were included in the propensity score. These variables included age, BMI, chronic obstructive pulmonary disease, dyslipidemia, peripheral artery disease, prior coronary stenting, CS with STEMI, SOFA, and VIS on the day of inclusion. Logistic regression was used for each patient to estimate the probability of experiencing 90-day all-cause mortality. To balance vasopressin and no vasopressin groups, weights were assigned to each patient using the stabilized inverse probability of treatment weighting (IPTW). Balance was assessed, with standardized mean differences (SMDs) lower than 10% considered acceptable [24].

Survival curves at 90 days were generated using the Kaplan–Meier method and compared between the two groups using the log-rank test (before weighting) and the Peto test (after weighting).

After accounting for mortality factors, we analyzed the reduction in NEE score in both groups using an ANOVA weighted test. Post hoc Bonferroni analysis was performed at four time points (day of inclusion, Day 1, Day 2, and Day 3).

The propensity score for SAE included prognostic variables related to SAE with a 10% significance level in univariate analysis. These variables encompassed age, atrial fibrillation, current smoking status, prior coronary stenting, CS with STEMI, SOFA score, and the NEE dose on the day of inclusion. For both 90-day mortality and the occurrence of SAE, hazard ratios (HRs) were estimated before and after weighting using Cox proportional hazards analysis.

To assess the consistency of our findings, we conducted a sensitivity analysis excluding patients who received ECMO. A propensity score was re-estimated, and IPTW was applied to this restricted cohort to adjust for baseline differences. Covariate balance was reassessed using SMDs, ensuring an acceptable balance threshold (< 10%). The association between vasopressin and 90-day mortality was then re-evaluated before and after weighting. Additionally, a re-estimated Kaplan–Meier survival curve was generated to compare survival outcomes between groups.

All tests were two-sided, and the threshold for statistical significance was set to *p* < 0.05. Statistical analysis was performed with R studio software for macOS (Version 2021.09.1+372) and its “dplyr,” “ggplot2,” “survminer,” “survival,” “hrbrthemes,” “tableone,” “ggeffects,” “WeightIt,” “cobalt,” “compareGroups,” “mice” and “epiR,” and “reshape2” packages.

## 3. Results

**Figure 1:**
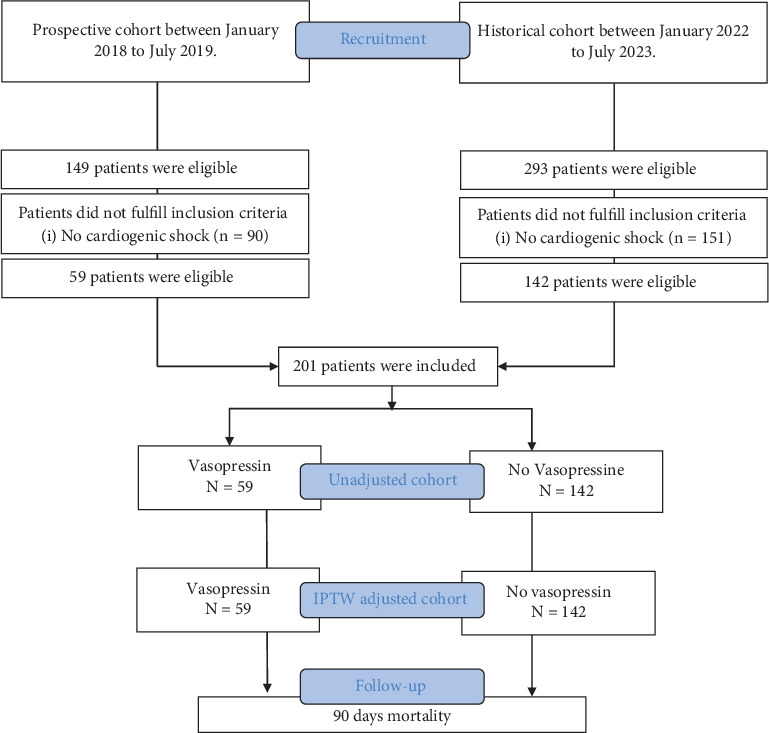
Flow chart of the study. IPTW, inverse probability of treatment weighting.

During the inclusion periods, 342 patients were eligible: 293 patients in the historical cohort and 149 in the vasopressin cohort (see Figure 1). Two hundred forty-one patients were excluded from the analysis because they had never experienced CS: 90 in the vasopressin cohort (*n* = 90/149, 60%) and 151 in the historical cohort (*N* = 151/239, 63%). Two hundred one patients with at least a Stage C of CS were finally included: 142 patients from the historical cohort (no vasopressin group) and 59 from the vasopressin cohort (vasopressin group).

The characteristics and clinical course of the population are summarized in Table 1. In the no vasopressin group, patients had a higher prevalence of chronic coronary artery disease (41/142, 29% vs. 9/59, 15%; *p* = 0.04) and a higher SOFA score (13 [10–16] vs. 12 [9–15]; *p* = 0.02) than those in the vasopressin group.

Additionally, CS was predominantly observed in the no vasopressin group following cardiac surgery (*N* = 91/149, 64% vs. *N* = 27/59, 46%; *p* = 0.04). In contrast, medical CS was more prevalent in the vasopressin group (*N* = 32/59, 54% vs. *N* = 51/142, 36%; *p* = 0.01), particularly in the context of septic cardiomyopathy (*N* = 7/32, 22% vs. *N* = 0/51; *p* = 0.001).

On the day of enrollment, patients in the vasopressin group had lower diastolic arterial pressure (55 ± 15 mmHg vs. 60 ± 11 mmHg; *p* = 0.02) than the no vasopressin group. Additionally, they exhibited higher NEE scores (0.72 [0.21–1.51] vs. 0.13 [0.07–0.34]; *p* < 0.001) and greater VIS values (141 [92–298] vs. 19 [10–41]; *p* < 0.001).

There was no difference for the use venoarterial (VA) ECMO support in the vasopressin group than in the no vasopressin group (*n* = 16/59, 27% vs. *n* = 45/142; 32%; *p* = 0.063). In patients with VA-ECMO, the NEE score was 0.26 (0.08–0.38) at the time of vasopressin introduction.

In the sensitivity analysis excluding ECMO patients, no association was found before weighting (HR = 0.96, 95% CI: 0.46–2.00; *p* = 0.93). After adjustment using IPTW (see Figure A1), vasopressin remained nonsignificantly associated with 90-day mortality (HR = 0.90, 95% CI: 0.33–2.47; *p* = 0.80). Moreover, the analysis of Kaplan–Meier curves showed no significant differences before (see Figure A2) and after weighting (see Figure A3).

There was no significant difference in the incidence of SAEs between the two groups (*n* = 57/142, 40% vs. *n* = 22/59, 39%; *p* = 0.88). The 90-day mortality (*n* = 31/59, 52% vs. *n* = 75/142, 52%; *p* = 0.97) was similar between the two groups. The 90-day survival curves, analyzed using Kaplan–Meier curves, did not reveal any difference between the two groups (Peto test with *p* = 0.91, see Figure A4).

### 3.1. IPW for the Endpoints

**Figure 2:**
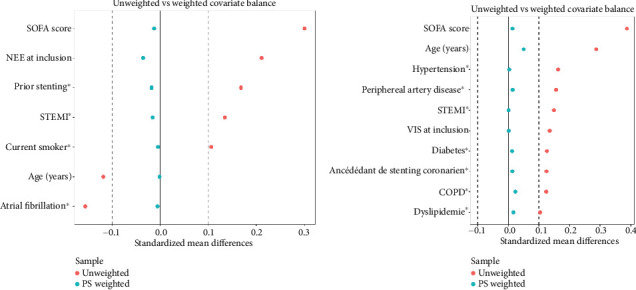
(a) Love plot of variable for the 90-day overall mortality. (b) Love plots for the SAE. SAE, serious adverse event.

Tables 2 and 3 summarize clinical variables associated with 90-day mortality and the occurrence of SAEs (DMS > 0.10) for the construction of the IPW. After IPW, for the assessment of 30-day mortality and occurrence of SAEs, the mean standard difference was < 10% for clinical variables. We also report the variables in a Love plot for 90-day mortality (Figure 2a) and the occurrence of SAEs (Figure 2b).

### 3.2. Association Between Vasopressin and the Endpoints

**Figure 3:**
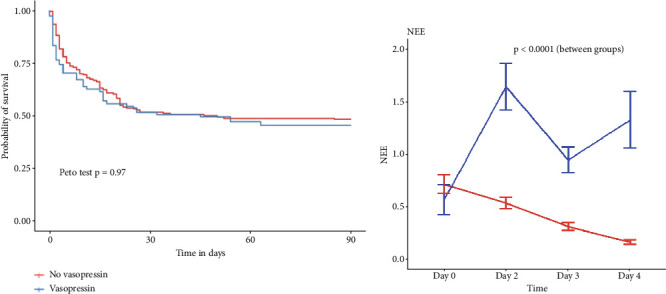
(a) Survival cumulative curves assessing 30-day overall mortality after weighting. (b) Evolution of NEE between the two groups after weighting on prognostic variables associated with 90-day mortality. NEE, norepinephrine equivalent score.

Before IPW, vasopressin was not associated with 90-day mortality (HR = 0.98 [95% CI 0.50–1.78]; *p* = 0.87). After weighting, vasopressin was not associated with 90-day mortality (OR = 1.10 [95% CI 0.56–2.17]; *p* = 0.77). After weighting, the analysis of survival curves using the Kaplan–Meier method found no difference between the two groups, as evidenced by a Peto test result of 0.92 (Figure 3a). Before weighting, vasopressin was not associated with the occurrence of an SAE (HR = 1.10 [0.56–2.17]; *p* = 0.77). After adjustment by propensity score, vasopressin was not associated with the occurrence of an SAE (HR = 0.91 [95% CI 0.35–1.83]; *p* = 0.61).

### 3.3. Evolution of NEE Score

In order to assess the vasopressor sparing effect of vasopressin, we compared the evolution of NEE score between the two groups via an ANOVA test weighted on variables associated with 90-day mortality (Table 4). After weighting, NEE was similar between groups (0.71 ± 1.04 vs. 0.56 ± 1.09; *p* = 0.58). However, the NEE course showed that NEE was, on average, 0.40 higher (95% CI = [0.14; 0.65]) in the vasopressin group (Figure 3). Additionally, the change in NEE varies between the two groups (*p* < 0.0001). The reduction in NEE over 3 days is higher in the no vasopressin group (0.71 [1.04–0.15, 0.26, *p* < 0.001]) than in the vasopressin group.

## 4. Discussion

Results of our retrospective study assessing the association between vasopressin and 90-day mortality in CS, using a propensity score approach, can be summarized as follows: (1) vasopressin was not associated with 90-day mortality before and after weighting, (2) 90-day mortality in the vasopressin group was 52%, (3) vasopressin showed no association with the occurrence of SAEs before and after weighting, and (4) vasopressin use did not lead to a significant decrease in NE doses.

### 4.1. Association Between Vasopressin and Mortality

In our study, vasopressin was not associated with mortality before and after propensity score weighting (HR = 1.10 [95% CI 0.56–2.17]; *p* = 0.77). These results are consistent with the VANCS study, which assessed vasopressin versus NE in vasoplegic and CS post-CPB (39% of patients were on dobutamine) [13]. In that study, vasopressin reduced the composite outcome of death and severe complications over 30 days compared to NE but did not affect mortality alone. In the VASST study, a prospective evaluation of vasopressin combined with NE versus noradrenaline alone, no difference in overall 28-day mortality was found [25]. However, the underlying pathological conditions were different, as VASST involved patients in septic shock, while our study focused on CS.

### 4.2. Incidence of Mortality and Vasopressin

In our study, the 90-day mortality rate among patients in the vasopressin group was 52%. This result aligns with the observed mortality rate, typically ranging from 40% to 60%, in CS [4, 26]. However, in a retrospective analysis evaluating the clinical impact of vasopressin on mean arterial pressure (MAP) in patients with CS, Nguyen et al. reported a higher 30-day mortality of 79% (*n* = 79/100) [27]. Notably, in that study, many patients were on VA-ECMO (*N* = 56/100; 56%) with a median NEE score at 1.44 (1.02–2.40) upon vasopressin initiation. In contrast, in our study, only 45 patients (31%) were on VA-ECMO, and the median NEE was 0.26 (0.08–0.38) at vasopressin introduction. It is possible that our patient population was in a less advanced state of CS at the time of vasopressin initiation compared to Nguyen et al.'s study.

### 4.3. Association Between Vasopressin and the Occurrence of SAEs

In our study, 22 SAEs were reported in the vasopressin group, including three ischemic strokes (5%), five digital ischemia (8.5%), seven gastrointestinal ischemia (12%), and seven myocardial infarctions (12%). Our findings closely resemble those of the VANCS study, which reported an incidence of 3% for ischemic strokes (*N* = 4/149), 1% for digital ischemia, 1% for mesenteric ischemia, and 11% for myocardial infarctions [13]. In the study by Nguyen et al., 20% of patients developed mesenteric ischemia, 10% experienced digital ischemia, and the authors reported no association between SAEs and vasopressin doses [27]. In our study, vasopressin was not associated with SAEs before and after propensity score weighting. These results are in accordance with those of major studies (VANCS [13] and VASST [25] studies.)

### 4.4. Vasopressin and NEE Reduction

Vasopressin is often part of catecholamine-sparing strategies that combine pharmacological and nonpharmacological treatments [28]. Recent clinical trials have suggested that vasopressin may be more effective than NE, and adding vasopressin can lead to a more rapid decrease in NE doses [25]. However, our study showed no significant reduction in the NEE score in the vasopressin group 3 days after initiation. Furthermore, in our study, the reduction in NEE dosage in the group without vasopressin was not associated with decreased mortality. It seems that the vasopressin group has a more profound vasoplegic state despite similar severity and outcomes. These results should be taken with caution. Current guidelines stress the importance of providing supportive care to restore organ perfusion, which involves initiating and adjusting vasoactive drugs to maintain hemodynamics, typically targeting a MAP of ≥ 65 mmHg [5]. However, determining the optimal MAP target in CS has been challenging due to limited data, as it has been derived from observational studies of septic shock. If the reduction in vasoactive drug doses indicates hemodynamic stability, its correlation with clinical outcomes remains uncertain. Moreover, like septic shock, CS is characterized by various pathophysiological alterations in microcirculation and ventriculoarterial coupling that contribute to organ dysfunction regardless of MAP level, necessitating specific treatments. It is necessary to develop clinical, biological, biochemical, or genetic markers to identify patients who would respond to different approaches to improve survival.

### 4.5. Vasopressin and the Vasopressor-Sparing Strategies

Vasopressin is now integrated into shock management, yet its role in CS remains unclear. Vasopressor-sparing strategies aim to reduce adrenergic pharmacological exposure and associated adverse effects, with several algorithms primarily developed for septic shock [29].

In our study, vasopressin did not significantly reduce NE requirements, particularly NEE, which aligns with prior findings suggesting limited efficacy in CS [27]. However, vasopressin may serve as an alternative to epinephrine, which is associated with increased mortality in CS patients [30]. No prospective randomized trials have evaluated vasopressin in this setting to date. While reducing vasopressor exposition could theoretically improve organ perfusion, high-quality evidence is lacking. Multicenter randomized control studies are needed to clarify its role, optimize vasopressor weaning strategies, and assess clinical outcomes in CS.

## 5. Limitations

Our study had limitations and biases inherent to all observational retrospective studies evaluating the effectiveness of pharmacologic treatment, even though the use of a propensity score seeks to reduce their impacts.

First, the retrospective design inherently limits the ability to define a strictly identical baseline time across patients. However, given the 90-day mortality trajectories, minor variations in inclusion timing are unlikely to have introduced a significant bias in mortality outcomes.

Second, patients in the vasopressin group had a more severe clinical profile and higher NEE at inclusion than those in the no vasopressin group, likely due to a delay in vasopressin initiation. In the no vasopressin group, patients were included 6 h after ICU-CTVR admission for CS, whereas in the vasopressin group, inclusion corresponded to the initiation of vasopressin. This suggests that vasopressin was selectively used in cases of therapeutic failure rather than as a systematic cotreatment.

Third, despite the application of the IPW method to adjust for baseline severity differences, residual confounding cannot be entirely ruled out. To address this potential bias, we conducted a sensitivity analysis excluding patients on ECMO, with the population weighted primarily based on low cardiac output syndrome etiology. This analysis yielded consistent results before and after weighting. However, the reweighting of the most critically ill patients may have attenuated the observed effect of vasopressin in this subgroup. Although the propensity score method was used to adjust for baseline differences, it cannot fully eliminate biases related to the initial decision to administer vasopressin, which was determined by the treating physician based on clinical judgment, highlighting the need for randomized controlled trials to generate more robust evidence on its therapeutic impact.

Additionally, unmeasured clinical variables may have influenced vasopressin administration, introducing potential selection bias. Nevertheless, even after adjusting for NEE and SOFA scores, we did not observe any trend toward a difference in mortality between the two groups. The timing of vasopressin administration may be a crucial factor, as late initiation may not be beneficial. As suggested by several authors, early administration could be preferable to better assess its impact.

Furthermore, some unmeasured confounders might have influenced the propensity score, such as left ventricular ejection fraction (LVEF) or cardiac output—both important prognostic factors in CS. These variables were missing in over 40% of cases in our study. However, the clinical impact of this limitation is likely minimal, as LVEF and cardiac output do not typically alter the classification of CS between Stages C and D, as opposed to earlier stages (A and B).

Regarding SCAI severity stratification, we included only CS cases classified as at least Stage C. However, precise classification of Stages D and E was limited by the absence of recorded SCAI scores at the time of inclusion, introducing a risk of misclassification. Additionally, dynamic severity changes within the first 24 h may have further impacted classification accuracy. Nonetheless, established severity markers such as NEE, SAPS II, SOFA, and VIS scores provide a robust assessment despite the absence of SCAI classification at admission.

## 6. Conclusion

In this retrospective study with a propensity score-weighted analysis on patients with at least Stage C of CS, the administration of vasopressin did not improve the clinical prognosis of patients at 90 days. Multicenter, randomized clinical trials in patients with CS are necessary to determine the usefulness of vasopressin in this specific setting.

## Figures and Tables

**Figure 4:**
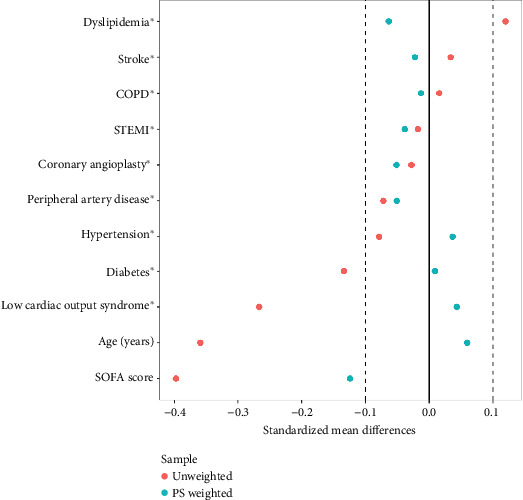
Love plot representing the standardized mean differences for covariates included in the propensity score model, before and after weighting. This allows assessment of covariate balance in the population without ECMO in relation to 90-day mortality.

**Figure 5:**
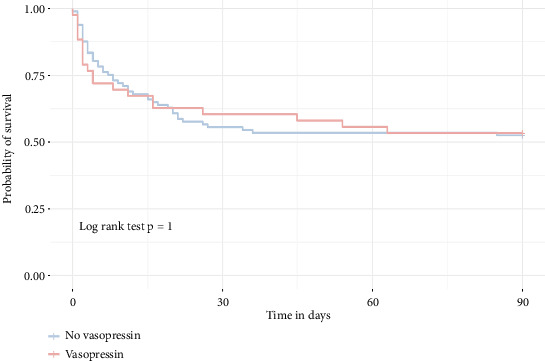
Kaplan–Meier survival curves depicting the unadjusted 90-day overall mortality in the population without ECMO. The plot illustrates baseline differences in survival between groups before propensity score weighting.

**Figure 6:**
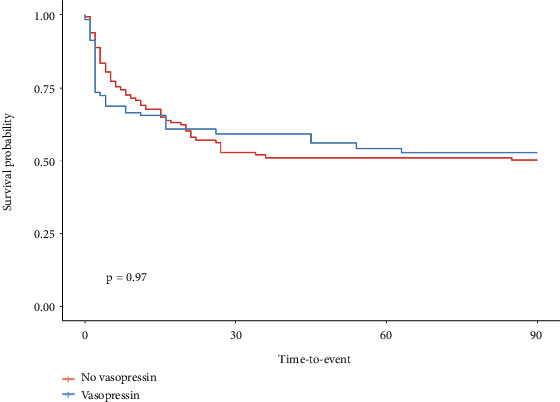
Weighted Kaplan–Meier survival curves assessing 90-day overall mortality in the non-ECMO population after applying inverse probability of treatment weighting (IPTW), demonstrating adjusted group comparisons.

**Figure 7:**
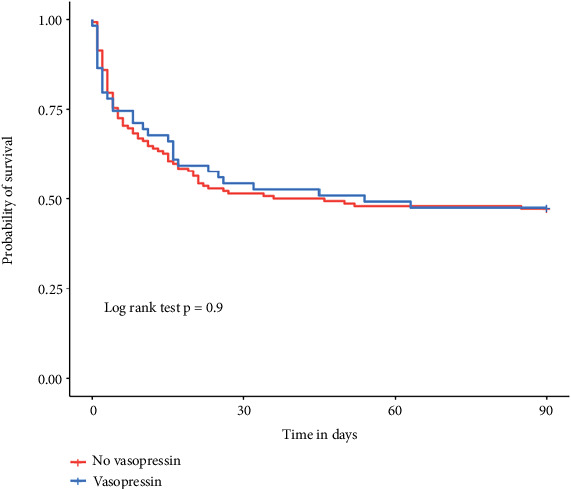
Kaplan–Meier survival curves for the overall study population prior to any statistical adjustment. This figure serves as a reference for baseline mortality trends before any weighting procedures.

**Table 1:** Baseline characteristics of the study population.

**Variables**	**No vasopressin (** **n** = 142**)**	**Vasopressin (** **n** = 59**)**	**p** ** value**	**SMD**
Age (years)	65 ± 13	62 ± 15	0.20	0.20
BMI (kg.m^−2^)	27.6 ± 5	28.3 ± 7	0.28	0.10
Female gender, *n* (%)	39 (27.5)	15 (25)	0.76	0.04
SOFA score	13 ± 3	12 ± 3	0.025	0.43
*Medical history, n (%)*				
Chronic coronary disease	41 (29)	9 (15)	0.04	0.33
Recent myocardial infarction	29 (21)	13 (22)	0.80	0.04
Peripheral vascular disease	20 (14)	7 (12)	0.67	0.006
Hypertension	87 (61)	36 (61)	0.97	0.005
Current smoker	33 (23)	17 (29)	0.41	0.12
Diabetes mellitus	34 (24)	9 (15)	0.17	0.22
Dyslipidemia	52 (37)	31 (52)	0.04	0.32
Chronic renal disease	18 (13)	6 (11)	0.62	0.08
Stroke	14 (10)	6 (11)	0.91	0.01
Atrial fibrillation	44 (31)	15 (25)	0.43	0.12
Chronic obstructive pulmonary disease	17 (12)	11 (19)	0.21	0.18
Prior cardiac surgery	9 (6)	5 (8)	0.58	0.08
Prior stenting				
*CS following cardiac surgery (n* = 118)	91 (64)	27 (46)	0.04	0.37
LCOS	76 (83)	23 (85)	0.95	
pMI	3 (3)	0	—	
Septic cardiomyopathy	12 (13)	4 (15)	0.85	
*Medical etiologies (n* = 83)	51 (36)	32 (54)	0.01	0.286
AMICS (*n* = 62)	44 (86)	18 (56)	0.04	—
STEMI	32 (72)	7 (39)	0.21	—
Nonischemic cause	7 (14)	7 (22)	0.42	—
Septic cardiomyopathy	0	7 (22)	0.001	—
*Biological data at inclusion*				
pH	7.30 ± 0.15	7.32 ± 0.12	0.19	0.19
Lactate (mmol.L^−1^)	4.7 ± 4.7	5.0 ± 5	0.71	0.06
Serum creatinine (*μ*mol.L^−1^)	134 ± 100	155 ± 72	0.18	0.19
Troponin Tc HS (ng.mL^−1^)	40, 410 ± 11, 200	36, 814 ± 13, 000	0.85	0.03
Hemoglobin (g.dL^−1^)	11.4 ± 2.3	11.3 ± 2.3	0.86	0.02
*Hemodynamic data at inclusion*				
Heart rhythm (bpm)	98 ± 22	102 ± 22.05	0.23	0.05
Systolic arterial pressure (mmHg)	103 ± 14	107 ± 25	0.28	0.35
Mean arterial pressure (mmHg)	71 ± 11	72 ± 15	0.61	0.35
Diastolic arterial pressure (mmHg)	60 ± 11	55 ± 15	0.02	0.51
*Vasopressor and dose at inclusion*				
Norepinephrine (*μ*.kg.min^−1^)	0.24 ± 0.36	1.14 ± 1.47	0.001	0.18
Dobutamine (*μ*.kg.min^−1^)	6.1 ± 6.2	5.48 ± 5.87	0.53	0.09
Epinephrine (*μ*.kg.min^−1^)	0.05 ± 0.14	0.09 ± 0.30	0.95	0.19
VIS score at inclusion	34 ± 46	128.3 ± 166.7	0.001	0.76
NEE score at inclusion	0.29 ± 0.46	1.23 ± 1.65	0.001	0.19
*Clinical course, n (%)*				
New onset of atrial fibrillation	37 (26)	21 (36)	0.17	0.21
Ventilator-associated pneumonia	39 (27)	22 (37)	0.17	0.21
Duration of mechanical ventilation (days)	8 ± 14	13 ± 32	0.28	0.19
Renal replacement therapy	77 (54)	38 (64)	0.18	0.20
VA-ECMO	45 (31)	16 (27)	0.63	0.24
*Serious adverse events, n (%)*	57 (40)	23 (39)	0.88	0.02
Ischemic stroke	15 (11)	3 (5)	0.21	0.20
Digital ischemia	6 (4)	5 (8.5)	0.23	0.17
Bowel ischemia	31 (22)	7 (12)	0.11	0.27
Myocardial infarction	11 (8)	7 (12)	0.35	0.14
*Outcomes*				
90-day mortality, *n* (%)	75 (52)	31 (52)	0.97	0.005
Length of ICU stay (days)	13 ± 16	16 ± 17	0.31	0.15
Length of hospital stay (days)	20 ± 24	20 ± 20	0.92	0.01

*Note:* Data are expressed in number, count (percent), and mean (±standard deviation).

Abbreviations: AMICS, acute myocardial infarction complicated by cardiogenic shock; BMI, body mass index; ICU, intensive care unit; LCOS, low cardiac output syndrome; NEE, norepinephrine equivalent; pMI, perioperative myocardial infarction; SMD, mean standardized difference; SOFA, sepsis organ failure assessment; STEMI, ST-segment elevation myocardial infarction; VA-ECMO, venoarterial extracorporeal membrane oxygenation; VIS, vaso-inotropic score.

**Table 2:** Imbalance of patient's characteristics before and after propensity weighting in the assessment of 90-day mortality.

**Variables**	**Before weighting**			**After weighting**		
	**No vasopressin (** **n** = 142**)**	**Vasopressin (** **n** = 59**)**	**SMD**	**No vasopressin (** **n** = 145**)**	**Vasopressin (** **n** = 45**)**	**SMD**
Age (years)	65 ± 13	62 ± 15	0.202	62 ± 15	63 ± 15	0.040
BMI	27.6 ± 5	28.3 ± 7	0.109	27.9 ± 5.7	27.4 ± 6	0.070
Hypertension	87 (61)	36 (61)	0.974	89 ± 61	32 ± 61	0.002
Dyslipidemia	52 (37)	31 (52)	0.325	57 (40)	23 (41)	0.014
COPD	17 (12)	11 (19)	0.186	20 (14)	8 (15)	0.031
Peripheral artery disease	20 (14)	97 (12)	0.066	17 (12)	5 (10)	0.090
SOFA score	13 ± 2	12 ± 2	0.472	12 ± 3	12 ± 3	0.002
Diabetes	34 (24)	9 (16)	0.220	30 (21)	10 (18)	0.083
Coronary angioplasty	32 (22)	7 (12)	0.286	27 (18)	8 (14)	0.085
VIS at Day 0	34 ± 46	128 ± 166	0.767	66 ± 93	68 ± 110	0.019

*Note:* Data are expressed in number, count (percent), and mean (±deviation standard).

Abbreviations: BMI, body mass index; COPD, chronic obstructive pulmonary disease; SMD, standardized mean difference; SOFA, sepsis organ failure assessment; STEMI, ST-segment elevation myocardial infarction; VIS, vaso-inotropic score.

**Table 3:** Imbalance of patients' characteristics before and after propensity weighting in assessing the occurrence of SAEs.

**Variables**	**Before weighting**			**After weighting**		
	**No vasopressin (** **n** = 142**)**	**Vasopressin (** **n** = 59**)**	**SMD**	**No vasopressin (** **n** = 145**)**	**Vasopressin (** **n** = 56** )**	**SMD**
Age (years)	65 ± 13	62 ± 15	0.202	65 ± 12	61 ± 16	0.286
Atrial fibrillation	44 (31)	15 (25)	0.120	44 (30)	11 (20)	0.230
Prior stenting	113 (80)	43 (73)	0.158	31 (22)	17 (30)	0.186
SOFA score	13 ± 2	12 ± 2	0.472	12 ± 3	12 ± 3	0.310
STEMI	32 (22)	7 (12)	0.282	35 (24)	8 (14)	0.247
NEE at Day 0	0.29 ± 0.46	1.23 ± 1.65	0.197	0.29 ± 0.44	1.30 ± 1.88	0.240
Current smoker	33 (23)	17 (29)	0.127	36 (25)	17 (31)	0.042

*Note:* Data are expressed in number, count (percent), and mean (±deviation standard).

Abbreviations: NEE, norepinephrine equivalent exposure; SAEs, serious adverse events; SMD, standardized mean difference; SOFA, sepsis organ failure assessment; STEMI, ST-segment elevation myocardial infarction.

**Table 4:** Evolution of NEE score between groups after weighting on prognostic variables associated with 90-day mortality.

	**No vasopressin group (** **N** = 150**)**	**Vasopressin group (** **N** = 54**)**	**p** ** value**
NEE at inclusion	0.71 ± 1.04	0.56 ± 1.09	*p* = 0.57
NEE at Day 1	0.52 ± 0.65	1.63 ± 1.70	*p* < 0.001
NEE at Day 2	0.30 ± 0.44	0.94 ± 0.93	*p* < 0.001
NEE at Day 3	0.15 ± 0.26	1.32 ± 2.07	*p* < 0.001

*Note:* Data are expressed in number and mean (±deviation standard).

## Data Availability

The data that support the findings of this study are available from the corresponding author upon reasonable request.

## Nomenclature

CS: cardiogenic shock
BMI: body mass index
CTVR: cardiothoracic vascular and respiratory
ECMO: extracorporeal membrane oxygenation
HR: hazard ratio
ICU: intensive care unit
IPTW: inverse probability of treatment weighting
NE: norepinephrine
NEE: norepinephrine equivalent
SAE: serious adverse event
SCAI: Society for Cardiovascular Angiography and Interventions
SMD: standard mean difference
SOFA: sepsis organ failure assessment
STEMI: ST-segment elevation myocardial infarction
VIS: vaso-inotropic score
